# Framework to describe constructs of academic emotions using ontological descriptions of statistical models

**DOI:** 10.1186/s41039-016-0029-1

**Published:** 2016-01-29

**Authors:** Keiichi Muramatsu, Eiichirou Tanaka, Keiichi Watanuki, Tatsunori Matsui

**Affiliations:** 1 Graduate School of Science and Engineering, Saitama University, 255 Shimo-Okubo, Sakura-ku, Saitama, Saitama 338-8570 Japan; 2 Faculty of Human Sciences, Waseda University, 2-579-15 Mikajima, Tokorozawa, Saitama 359-1192 Japan

**Keywords:** Ontology, Academic emotions, Boredom, Construct

## Abstract

Many studies have been conducted during the last two decades examining learner reactions within e-learning environments. In an effort to assist learners in their scholastic activities, these studies have attempted to understand a learner’s mental states by analyzing participants’ facial images, eye movements, and other physiological indices and data. To add to this growing body of research, we have been developing the intelligent mentoring system (IMS), which performs automatic mentoring by using an intelligent tutoring system (ITS) to scaffold learning activities and an ontology to provide a specification of learner’s models. To identify learner’s mental states, the ontology operates on the basis of the theoretical and data-driven knowledge of emotions. In this study, we use statistical models to examine constructs of emotions evaluated in previous psychological studies and then produce a construct of academic boredom. In concrete terms, we develop ontological descriptions of academic boredom that are represented with statistical models. To evaluate the validity and utility of the descriptions, we conduct an experiment to obtain subjective responses regarding learners’ academic emotions in their university course and describe them as instances on the basis of the ontological descriptions.

## Introduction

During the last two decades, studies have been conducted that examine semiconscious behaviors of learners participating in e-learning environments by observing and analyzing facial images, eye movements, and other physiological indices. Analysis of data obtained from such examinations enables researchers to understand the various mental states of learners, such as “confidence” and “confusion” (Arroyo et al. 2009; Muldner et al. 2009). In addition, studies have used the intelligent tutoring system (ITS) to evaluate structural features of the knowledge that learners possess. Knowledge of learners’ behaviors such as eye movements, facial expressions, keyboard actions, and speech is helpful for building intelligent systems that support learning from the aspects of both knowledge and mental states. Eye movement information is important data that is generally useful for realizing human mental processes. Such data from eye movement enables the detection of learners’ mental states in greater detail (Muldner et al. 2009; Ueno and Nagaoka 2005). Facial expressions are used in assessment of learners’ affective states especially focused on boredom, confusion, and frustration (D’Mello et al. 2009) and in preliminary study to detect, monitor, and record emotions during learning sessions (Azevedo and Strain 2011). Furthermore, keyboard action and speech during learning were also used as resources to detect learners’ affect. Alepis et al. (2008) found relationships between such the resources and positive/negative feeling.

On the basis of these studies, researchers have proposed and have been developing an affect sensitive pedagogical agents (Arroyo et al. 2011; Sarrazfadeh et al. 2014) and an intelligent mentoring system (IMS) that supports learning activities from both knowledge and mental states (Kojima et al. 2012; Muramatsu et al. 2012; Muramatsu et al. 2013). One of its main characteristics is the diagnostic function of the learner model considering the mental states of learners. Because mental states can instantly change in a short activity (e.g., solving of a single problem), the IMS is required to monitor learners at all times and to give feedback based on diagnosis. The IMS provides integrative learning support including real-time estimation of learners’ mental states and a selection of ways to support learners, in addition to a diagnosis of learners’ knowledge structures and determination of teaching strategies provided by the ITS.

**Fig. 1:**
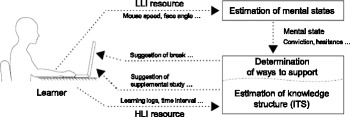
Skeleton framework of intelligent mentoring system

Figure 1 shows a skeleton framework of the IMS, in which data from interactions between users and the system are captured according to two levels of cognitive activity: high-level interactions (HLI) and low-level interactions (LLI). The HLI is explicitly accompanied by user awareness and is consequently illustrated by a data resource, which is sampled in large grain sizes. The data resources refer to objects that can be recorded or sensed by the systems. In an e-learning environment, for example, the operations of learners who take a multiple-choice test using mouse clicks are regarded as HLI resources, when the e-learning system captures their operations as behavioral data. On the other hand, LLI is not always accompanied by user awareness and is consequently illustrated by a data resource, which is sampled in very small grain sizes. For example, learning logs, time interval of operation (Nakamura et al. 2003), and required time for learning (Ueno and Nagaoka 2005) can be former data resources, and moving speed of a mouse, face or posture angle of learners, and gaze position (Nakamura et al. 2008) or eye movement of a learner can be latter data resources. Focusing on LLI resources, which are task-oriented and independent from knowledge structures of specific study domains, the IMS aims to estimate learners’ mental states from their unarticulated and semi-conscious behaviors.

The IMS’s function to determine ways to support learners is required to integrate information about a knowledge structure estimated from HLI resources (this corresponds to the function of the ITS) and mental states estimated from LLI resources. To fulfill the requirement, Muramatsu et al. (2012) developed an ontology that provides descriptions of the relationships among LLI resources and the mental states of learners. These ontological descriptions are based on specific tasks performed by learners, which are independent of the knowledge structures examined within specific learning domains. Muramatsu et al. (2013) expanded the ontological descriptions pertaining to mental states based on concepts of academic emotions, which were proposed by Pekrun et al. (2002). The academic emotions are defined as emotions of a student experienced in academic settings such as class-related, learning-related, and test-related situations and are characterized with subjective control and value perceived by learners in the control-value theory (Pekrun 2006). These descriptions help to clarify relationships between academic emotions and subjective attributes that perform the role of subjective control or value in accordance with the control-value theory. Their ontology effectively illustrates how academic emotions are formed during the co-occurrence of control and value, and it has helped researchers interpret learners’ mental states on the basis of LLI resources in the IMS.

The ontology would be a systematical reference used as building blocks of the IMS. When the IMS identifies the mental state of learners, an analysis of LLI resources would depend on the contexts of learners in academic activities. In line with the control-value theory, the IMS does not always directly identify learners’ mental states as a result of using LLI resources. That is, academic emotions can be identified, by control and value that are estimated by the LLI resources, as distinct from the direct estimation of other mental states including emotions. Moreover, an object in an academic activity, on which the learner is focusing when an academic emotion occurs, would also be identified by the use of resources, including those of the LLI, in the IMS. Therefore, it is important for the interpretation of learners’ mental states to clarify how their academic emotions are defined by control, value, and focused object and to build a framework to describe their construct. Once the constructs of academic emotions are collected as an ontology, the IMS is capable of supporting learners in various contexts.

However, the descriptions provide insufficient detail to identify subcategories where academic emotions are observed in practical situations. The subcategories derive from experiments that measure emotions using rating scales and statistical analyses of the measured data. To implement the IMS, ontological descriptions about academic emotions should include both data-driven and theoretical knowledge. Therefore, this study makes a conceptualization of constructs of an academic emotion by conceptualization of statistical models such as the factor analysis model, which is often used in psychological research. Some research tried to categorize emotions in general situations with ontology, for example, Arellano et al. (2009) built an Event Ontology to define categories of emotions related to actions for generating an emotional state in virtual characters. However, academic emotions have not been ontologically systematized with the exception of the ontological descriptions pertaining to academic emotions (Muramatsu et al. 2013). Therefore, we expanded the descriptions in the current study. Specifically, we introduce the structure of rating scales that express psychological attributes as representations and specify relationships among variables that represent the psychological attributes in statistical models. Finally, we demonstrate ontological descriptions of constructs of an academic emotion.

## Emotions in academic settings

In psychology, learner emotions, specifically within the context of classroom instruction and achievement, are referred to as academic emotions (Pekrun et al. 2002). Emotions related to achievement are defined as achievement emotions and are measured by using the achievement emotions questionnaire (Pekrun et al. 2011). This questionnaire consists of scales related to nine emotions: enjoyment, boredom, anger, hope, anxiety, hopelessness, pride, relief, and shame. These nine emotions can be subdivided into two types according to their object focus that means the focus of attention when an emotion is produced: (1) activity emotions, which pertain to ongoing achievement-related activities, and (2) outcome emotions, which concern the outcomes of these activities. Enjoyment, boredom, and anger constitute activity emotions. The outcome emotions include prospective outcome emotions such as hope, anxiety, and hopelessness, as well as retrospective outcome emotions such as pride, relief, and shame.

Academic emotions are explained by referring to the control-value theory proposed by Pekrun (2006). This theory describes emotions as sets of interrelated psychological processes composed primarily of affective, cognitive, motivational, and physiological dimensions (Pekrun et al. 2011). The theory appraises subjective control and value. The appraisal of subjective control relates to perceived control of achievement-related actions and outcomes. By contrast, the appraisal of subjective value pertains to the subjective importance of achievement-related activities and outcomes.

In e-learning environments, learning materials such as multiple-choice tests are considered as “object focuses,” and activity emotions such as enjoyment, boredom, and anger can arise in such settings. For example, when a learner’s mental states are estimated as “interesting” and “comprehending,” enjoyment is expected to be the academic emotion experienced. In this situation, the quality of “interesting” has a subjective value, which includes a quality value of positive or negative, because subjective evaluation on the quality of “interesting” correlates to a positive/negative affection (Acee et al. 2010). However, when an activity involves learning material that lacks incentive value, whether positive or negative, boredom is the expected result. The incentive value of an activity may depend on the control that is perceived by the learner (Pekrun 2006).

According to research on the construct of academic boredom, a learner’s perceptions of boredom also represent a situation-dependent construct (Acee et al. 2010). Specifically, over-challenging situations lead learners to either “task-focused” or “self-focused” boredom, while under-challenging situations lead to general boredom. The research of Acee et al. (2010) measures learners’ emotions using the academic boredom scale (ABS). The ten items in ABS (ABS-10) consist of unipolar scales that correspond to ten psychological attributes, which are listed as follows: “want something else,” “tired of activity,” “impatient,” “frustrated/annoyed,” “apathetic,” “nothing to do,” “activity dull,” “repetitive,” “wonder why doing this,” and “useless/unimportant.” As a result of a factor analysis of data related to under-challenging situations, all items in the ABS-10 scale were correlated to general boredom. By contrast, a factor analysis of data related to over-challenging situations correlated five psychological attributes (“want something else,” “tired of activity,” “impatient,” “frustrated/annoyed,” and “apathetic”) to self-focused boredom. The other five attributes (“nothing to do,” “activity dull,” “repetitive,” “wonder why doing this,” and “useless/unimportant”) were correlated to task-focused boredom. Because the variables derived from these factor analyses yield psychosocial attributes measured using rating scales, the relationships among them provide a construct of academic emotions and indicate that the boredom can be divided into subcategories.

For researchers developing an intelligent system such as IMS, it is important to share knowledge about constructs and subcategories of academic emotions because the learners’ mental states including academic emotions are to be predicted by data from learners’ behaviors such as LLI resource data. In concrete terms, the constructs and subcategories provide criteria or classes of mental states estimated by behavioral data.

## Method for ontology development

### Ontological engineering

Ontological engineering is a field of computer science that supports the systematic description of knowledge. From this knowledge-based perspective, “ontology is defined as a theory (system) of concepts/vocabulary used as building blocks of an information processing system” (Mizoguchi et al. 1995). Ontologies are classified into two types according to the nature of the knowledge described (Mizoguchi 2003). One is called *domain ontology*, which describes domain knowledge, and the other is *task ontology*, which describes knowledge about processes. In the current study, we aim to build a domain ontology to describe the static structure of academic emotions which occur in learning processes.

#### Role concept

**Fig. 2:**
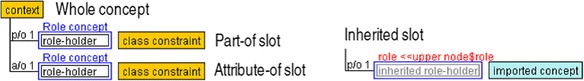
Legend of nodes and slots in Hozo ontology editor

In the Hozo ontology editor (http://www.hozo.jp/), which is an ontology development environment, each node represents a whole concept and contains slots that represent part-of or attribute-of relations (Fig. 2). Hozo helps to describe role concepts wherein a role depends on the contents of each whole concept. For example, a teacher’s role is played only in the context of school. Every slot thus has a role within a whole concept that implies a context. In the context, a class of instances that can play a role is defined by a class constraint and is called a role holder (Kozaki et al. 2000). In this way, the role concept distinguishes between concepts within different contexts. Inherited role holders and class constraints imported from other ontologies are shown in the right half of Fig. 2.

#### Top-level ontology

Mizoguchi (2010) constructed a top-level ontology based on the role concept theory known as “Yet Another More Advanced Top-level Ontology (YAMATO; http://download.hozo.jp/onto_library/upperOnto.htm).” On the basis of YAMATO, an entity is divided into three classes: physical, abstract, and semi-abstract. Although instances of a physical class require three-dimensional (3D) space and time to exist, instances of an abstract class require neither. Instances of a semi-abstract class require only time to exist, and the class contains mind, representation, content, and a representation form.

Representations such as novels, poems, paintings, music, and symbols are distinguished from their propositions and forms of representation (Mizoguchi 2004). A class of representation is further divided into two representations: primitive and composite. The composite representation has one or more part-of slots, which indicates that a subsidiary role is played by a representation. The representation contains part-of slots that indicate a content role played by a proposition and a form role played by a representation form. The proposition is divided into two classes: representation-primary and representation-secondary. For example, “content of a piece of music” and “content of a novel” are examples of the former, and “content of a fact recognized by a human” is an example of the latter. These classes necessarily depend on their representation. However, instances of a representation-secondary class, such as facts, data, and thoughts, indicate the original content that should be represented. For example, a fact is designated as an event that exists before it can be recognized and expressed as a representation. In this sense, the process of human recognition, which necessarily includes sensations and perceptions, belongs to the representation-secondary class.

YAMATO’s main features are definitions of qualities and quantities, their representations, and descriptions of their interrelationships in other top-level ontologies. Attributes of entities are represented as qualities that are composed of quality values. A quality value belongs to a “categorical” class, and a quantity contains a quantitative quantity and a qualitative quantity. A quality is divided into a property and generic quality, with the property being an abstraction of the generic quality but possessing a quality value. The generic quality is divided into “intrinsic generic quality” and “accidental generic quality.” A subclass of intrinsic generic quality is basic generic quality, which contains “quantitative generic quality” and “qualitative generic quality.”

In YAMATO, the representation of a quality is distinguished from a real quality which exists with an entity. Therefore, representations of qualities and quantities are defined as transformations of a real quality through an “action to measure.” The measure contains a part-of slot that indicates a “result” role played by a primitive representation. A quality measurement is defined as a role-holder performed by a proposition in a content role subslot of the result role slot. Through measurements, the data are approximations of real qualities, and a quality value representing a true value is independent of any measurements. Therefore, representations of a quality must be distinct from representations of a quality obtained through measurements (Masuya et al. 2011).

### Concepts for describing mental states

**Fig. 3:**
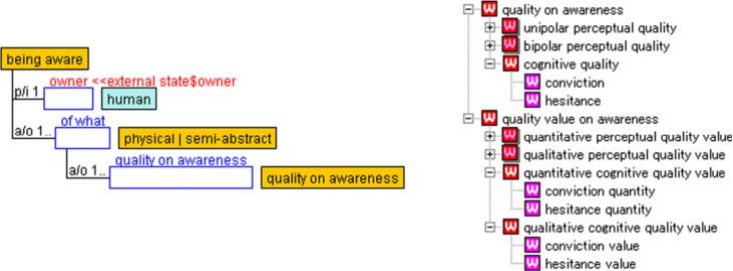
State of “being aware” and quality on awareness

We have partially expanded YAMATO to describe a subjective evaluation that is regarded as an expression of a psychological quantity. More precisely, it is defined as a representation of quality (defined in YAMATO) based on a doer’s awareness (Muramatsu et al. 2011). Doer’s awareness is described as a state of “being aware” (Fig. 3), which is defined as a subclass of “external state” in YAMATO. Objects of awareness are represented by “of-what” role-holders played by a physical or a semi-abstract. A subslot of of-what is “cognitive quality” played by “quality on awareness” represents a psychological quality that a doer subjectively feels.

The state of “being aware” indicates two types of consciousness. According to Baruss (1987), consciousness is defined as “all subjective awareness characterized by intentionality, and the explicit knowledge of one’s situation, mental states, or actions evidenced behaviorally.” Subjective awareness is referred to as subjective consciousness, that is, “the stream of thoughts, feelings, and sensations that occur for a person,” and explicit knowledge is referred to as behavioral consciousness (Baruss 2000). The latter is generated by the operationalization of the former, according to the literature. That is, behavioral consciousness is defined on the basis of operations that infer other people’s consciousness during objective studies. Thus, the state of “being aware” is defined under the “external state” in YAMATO in the sense of behavioral consciousness and is defined to have a reference to “quality on awareness” of an object which plays an “of-what” role in the sense of subjective awareness.

Qualities that exist on awareness and their values are sharply distinguished from physical qualities and their values that are defined in YAMATO. Figure 3 shows the hierarchy of “quality on awareness” and “quality value on awareness.” Learners’ psychological qualities such as conviction and hesitance are defined as a subclass of “cognitive quality” under the quality on awareness. For example, “conviction” has two “referring to” slots: one is played by “conviction quantity” and the other is played by “conviction value.” The conviction quality is a subclass of “quantitative cognitive quality value,” and the conviction value is a subclass of the “qualitative cognitive quality value.” Both quantitative and qualitative cognitive quality values are defined under “quality value on awareness.”

### Subjective measurement

**Fig. 4:**
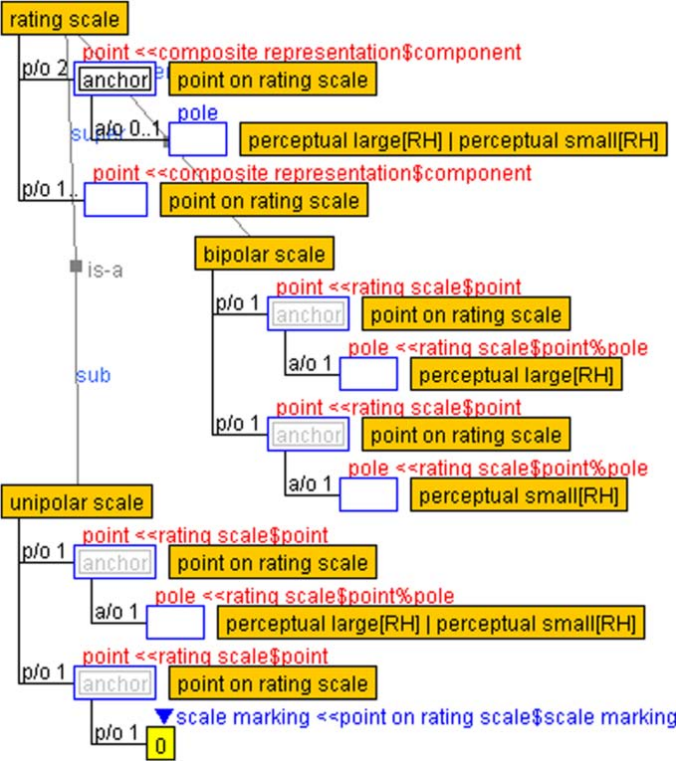
Rating scale and its points

In psychometric methods that use rating scales, subjective evaluations of emotions are often expressed as points on a scale. The *rating scale* and *point on rating scale* are displayed in Fig. 4. A point on the rating scale has a form slot that is filled by a *word* or *pictogram* and contains an additional slot in which a number represents a scale marking. The rating scale is a composite representation that consists of multiple points. Two points are considered *anchor* role-holders in which a *pole* subslot indicates a perceptual large or small point.

Semantic differential scales contain adjective pairs that represent perceptual qualities, each of which indicates large or small perceptual quality values. Thus, the relationship of magnitude among perceptual quality values can be defined through the rating scale. Furthermore, unipolar and bipolar scales, defined as subclasses of the rating scale, contain unipolar and bipolar perceptual qualities, respectively. For example, subjective evaluations of emotions in academic settings such as easy/difficult, boring/interesting, confused/comprehending, and tired/concentrating are specified as attributions on awareness and are represented on the rating scale. That is, subjective evaluation can be regarded as transforming the subjective quality on the doer’s awareness into a representation. In case of an adjective pair, “easy” and “difficult,” the former plays a “point” role and the latter plays the other “point” role.

### Mathematical models and data representations

**Fig. 5:**
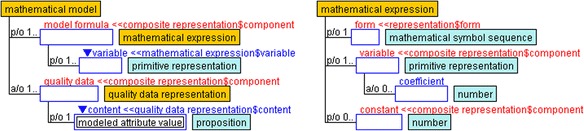
Mathematical model and its components

**Fig. 6:**
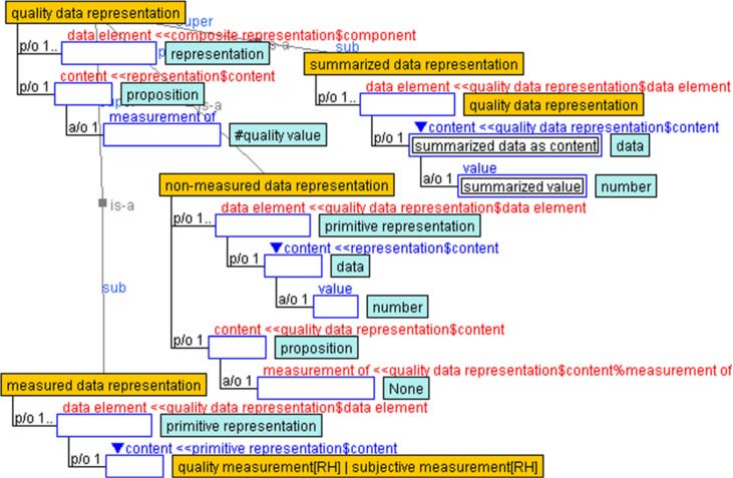
Mathematical and quality data representations

To show relationships between the measured data of emotions in a subjective way, statistical models are often adopted in psychological research (e.g., Linnenbrink-Garcia et al. 2011; Nett et al. 2011). In this study, we used unique mathematical models (Fig. 5) as well as mathematical and quality data representations (Fig. 6), which are defined under the composite representation of YAMATO. The *mathematical model* contains a *mathematical expression* slot and one more *quality data* slot inherited from the *composite* slot in the composite representation. Each role of the slots uses a mathematical expression and *quality data representation*. In the mathematical model, the content of the quality data is defined as a *modeled attribute value*, while the mathematical expression is composed of multiple *variable*s inherited from the *component* slot and *constant* slot. The variable role contains a representation and coefficient performed by a number defined as a subslot.

The quality data representation contains multiple data element role slots performed by other representations. The content role slot is played by the data, and the measurement of the subslot indicates the derivation. The quality data representation is divided into measured data representation, non-measured data representation, and summarized data representation. In the measured data representation, the content is performed by the quality measurement or subjective measurement, which indicates quality value as a proposition. However, the content of the non-measured data representation, such as factor scores and principal component scores, exists only in mathematical models. The summarized data representation is composed of data elements played recursively by the quality data representation, and its content is regarded as summarized data as content, which represents a summarized value such as an average.

The ontological description of the mathematical model indicates a set of formulas that contain some variables and data that are assigned to the variables. That is, the description focuses only on the correspondence between variables and data. For example, the model of factor analysis that is often adopted in educational research consists of multiple formulas where the objective and explanatory variables indicate measured data and factor score data, respectively. The measured raw data and averaged data are represented as “measured data representation” and “summarized data representation,” respectively, on the basis of the ontology. On the other hand, the factor score data is represented as “non-measured data representation.”

### Statistical models

**Fig. 7:**
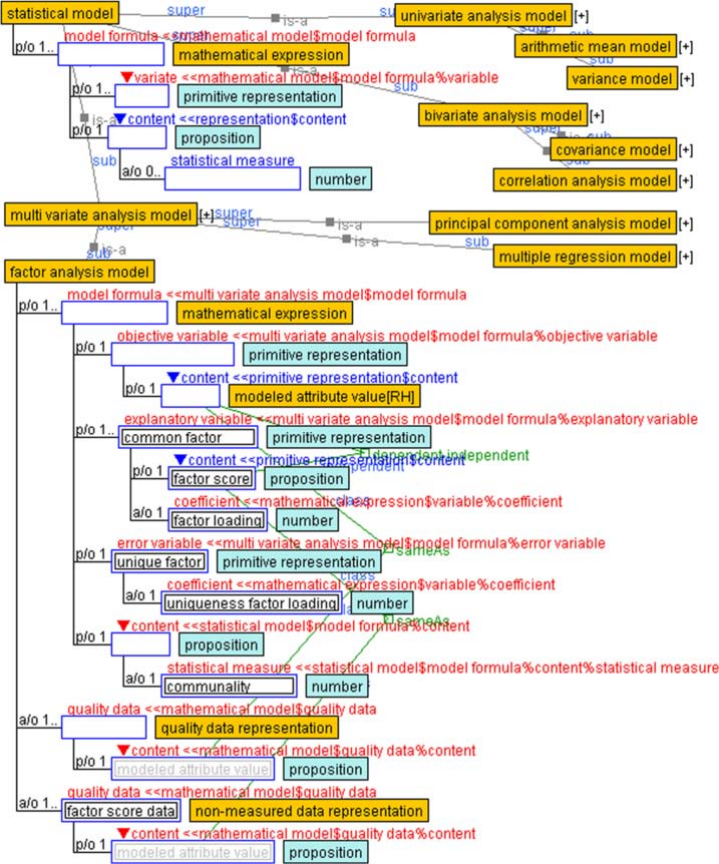
Statistical model and subclasses

Figure 7 displays the hierarchy of statistical models and their subclasses. The statistical model is defined under the mathematical model. Its subclasses comprise a univariate analysis model, bivariate analysis model, and multivariate analysis model by cardinality of the quality data representation slot. The univariate and bivariate analysis models use summary statistics such as arithmetic mean, variance, covariance, and correlation. Multivariate analyses such as multiple regression, factor analysis, and principal component analysis are defined as subclasses of the multivariate analysis model. Objective and explanatory variables are described in a model formula slot and have a “dependent-independent” link to indicate their correspondences. The data to be assigned to the variables is described by a “same as” link between the content slots of variables and the data representations.

## Description for construct of academic emotions

### Attributes referred to by variables

Ontological descriptions introduced in the previous section help to specify the construct of academic emotions, which are analyzed in psychological studies. As mentioned in “Emotions in Academic Settings” section, the results of a factor analysis conducted by Acee et al. (2010) indicate that academic boredom is comprised of multidimensional and situation-dependent constructs. The result is summarized as follows. First, some items on the ABS-36, which is built in the same literature (Acee et al. 2010), are correlated to negative and positive values. Second, all items on the ABS-10 are correlated to general boredom in under-challenging situations. Third, in over-challenging situations, five items that indicate “want something else,” “tired of activity,” “impatient,” “frustrated/annoyed,” and “apathetic” are correlated to self-focused boredom, whereas the remaining five items indicating “nothing to do,” “activity dull,” “repetitive,” “wonder why doing this,” and “useless/unimportant” are correlated to task-focused boredom.

Before describing the structure obtained by factor analysis, we clarified attributes referred to by variables used in the analysis. The ABS-10 consists of ten items representing ten psychological attributes: “want something else,” “tired of activity,” “impatient,” “frustrated/annoyed,” “apathetic,” “nothing to do,” “activity dull,” “repetitive,” “wonder why doing this,” and “useless/unimportant.” These psychological attributes are ontologically described as qualities on awareness, introduced in Fig. 3.

### Relationships between variables in statistical models

**Fig. 8:**
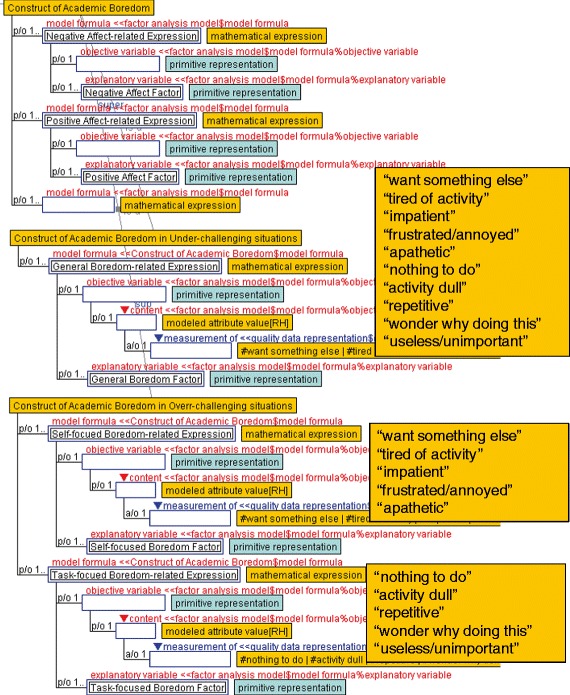
Construct of boredom defined under a statistical model

We described a construct of boredom, an academic emotion, by using ontological descriptions of statistical models to specify the construct of academic boredom (Fig. 8). In the figure, the *Construct of Academic Boredom* illustrates relationships among psychological attributes pertaining to boredom and is defined as a subclass of the factor analysis model (shown in Fig. 7). Their model formulae given in mathematical expressions (shown in Fig. 5) are defined as *Negative Affect-related Expression* and *Positive Affect-related Expression* role holders, which indicate relations between object variables and factors. The object variables contain a content slot used by a modeled attribute value, which is defined as a proposition of a quality data representation shown in Fig. 6. This means that the modeled attribute value refers to a quality value measured with a rating scale (shown in Fig. 4). Therefore, correlations between some items of the ABS-10 and negative/positive values are adequately described.

The constructs of boredom in under- and over-challenging situations are represented as subclasses of the *Construct of Academic Boredom* (Fig. 8). In the *Construct of Academic Boredom in Under-challenging situations*, the modeled attribute value that is correlated to the *General Boredom Factor* refers to a quality value measured with a rating scale. Types of qualities are specified by the role player in the “measurement of” role, and attributes playing roles in the measurement of slot are the qualities on awareness. For example, a quality measured by the ABS-10 such as “want something else,” “tired of activity,” or “impatient” can play that role. Similarly, modeled attribute values in the *Construct of Academic Boredom in Over-challenging situations* also refer to qualities measured by the ABS-10. As a result, objective variables that indicate modeled attribute values measured by specific rating scales and factors are organized as sets of model formulae.

## Discussion

In this section, we discuss the utility of the ontological descriptions proposed in the previous section through demonstration. First, we conducted an experiment to obtain data of subjective responses with the ABS-10.

Second, we show an example of handling actual data and interpreting a construct of boredom, which can be adopted by the IMS to discuss the utility of the descriptions.

### Experiment to obtain data of subjective responses

We conducted an experiment to collect subjective responses about academic boredom according to a previous study (Acee et al. 2010). Participants (seven graduate students: six males and one female) of this experiment were asked to answer each items of ABS-10 by considering two different situations: under-challenging and over-challenging. A nine-point Likert-type rating scale ranging from 1 “Not at all” to 9 “Extremely” was used. The items of the ABS-10 were shown to the participants with Japanese translation in addition to the original English statement. The instructions and items are listed in Tables 1 and 2.

**Table 1:** ABS-10 instructions

Instructions
Think of a situation in which you found academic activities too difficult and too challenging, in that it was hard to understand or too much work. The following questions pertain to accompanying thoughts and feelings in that situation. Please indicate what extent did you think or feel in the situation, by using nine-point scale from 1 “Not at all” to 9 “Extremely.”
Think of a situation in which you found academic activities too easy and not challenging, in that it was easy to understand and not much work. The following questions pertain to accompanying thoughts and feelings in that situation. Please indicate what extent did you think or feel in the situation, by using nine-point scale from 1 “Not at all” to 9 “Extremely.”

This instruction was originally used by Acee et al. (2010)

**Table 2:** ABS-10 item list

Items
In that situation, to what extent did you have nothing to do or think about?
In that situation, to what extent did you find the activity dull?
In that situation, to what extent did you feel it was repetitive?
In that situation, to what extent did you wonder why you were doing this?
In that situation, to what extent did you feel it was useless and unimportant, that you were wasting your time?
In that situation, to what extent did you want to do something else?
In that situation, to what extent did you get tired of the activity?
In that situation, to what extent did you become impatient?
In that situation, to what extent did you become frustrated or annoyed?
In that situation, to what extent did you feel apathetic, not wanting to do anything?

These items were originally used by Acee et al. (2010)

We analyzed data from the completed questionnaires of all seven participants. The mean and standard deviation of each item ranged from 2.9 to 7.9 and from 0.83 to 2.89, respectively. Next, we calculated Cronbach’s coefficient alpha to confirm the construct of academic boredom. In concrete terms, we examined whether the data indicates a one-factor structure, which indicates under-challenging situations, or a two-factor structure, which indicates over-challenging situations, according to the literature (Acee et al. 2010). The items related to self-focused boredom were “want something else,” “tired of activity,” “impatient,” “frustrated/annoyed,” and “apathetic.” The items related to task-focused boredom were “nothing to do,” “activity dull,” “repetitive,” “wonder why doing this,” and “useless/unimportant.” Thus, all items are simply separated into the two item sets. In under-challenging situations, it is assumed that a set of all items indicates an alpha value that is higher than both of the alpha values of items related to self-focused boredom and items related to task-focused boredom, according to a previous study by Acee et al. (2010). On the other hand, in over-challenging situations, it is assumed that the alpha value of item sets indicate that self- and task-focused boredom is higher than the value of the all items according to that study. Table 3 shows the calculated alpha values. The alpha value of the item set related to self-focused boredom was higher than all items in over-challenging situations. On the other hand, the alpha value of all items was higher than task- and self-focused boredom in under-challenging situations. As a result, the assumption that supports the proposition of the previous study was confirmed.

**Table 3:** Item sets and alpha values

Item sets	Alpha values
All items in over-challenging situations	0.833
Items related to task-focused boredom in over-challenging situations	0.748
Items related to self-focused boredom in over-challenging situations	0.860
All items in under-challenging situations	0.852
Items related to task-focused boredom in under-challenging situations	0.748
Items related to self-focused boredom in under-challenging situations	0.674

On the basis of the assumption, it is easily found which over- or under-challenging situations fit a set of items given to a participant, by a simple calculation. In concrete terms, which situations fit the given data are identifiable by comparing the sum of the squared deviations (total sum of squares (TSS)), calculated from all items to one from the separated item sets (self-focused and task-focused) for each situation, which is represented as a ratio between TSS of all items and one of the separated item sets. We found that the ratio in the over-challenging item set was higher than one in the under-challenging situation for all persons in the comparison.

In developing the IMS, this kind of data handling will be adapted to interpret mental states from LLI resource data. That is, estimating the subjective response of ABS-10 by using LLI resources is one method to interpret overall academic boredom. According to the current experiment, we confirmed that subjective responses relating to academic boredom are categorized into two types of boredom, which is consistent with the previous study and ontological descriptions. Therefore, the estimation of such subjective responses by LLI resources provides useful information for interpreting academic emotions. Whereas more complex calculations will be used in an actual development for practical settings, we hold this simple handling as an example to discuss the utility of the ontological descriptions.

### Utility of the ontological descriptions

As mentioned in the previous subsection, an introduced way of data handling is too simple to implement the mentoring function in IMS. However, we limit the discussion to the example because developing ways to handle data is a concern of other research. That is to say, we do not discuss how subjective responses pertaining to boredom that is measured by rating scales such as ABS are estimated by LLI resource data, but how ontological descriptions help to interpret the construct of boredom, which is known and classified according to previous studies. As mentioned above, we confirmed that subjective responses are easily categorized into two types of boredom based on the construct implemented in the ontological descriptions. Therefore, we discuss the utility of the ontological description by focusing on the process after estimation of these subjective responses in the IMS.

### Estimation of subjective responses pertaining to boredom

**Fig. 9:**
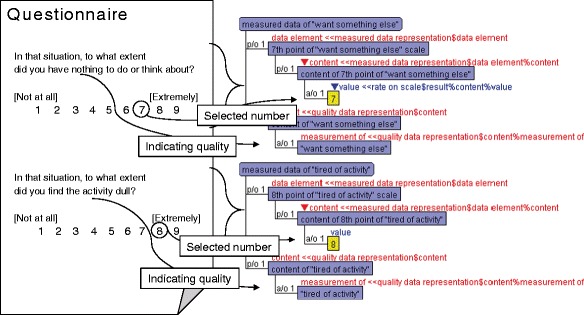
Correspondences between instance of data representations and real questionaire

On the basis of our ontological descriptions, the collected data in the previous subsection are represented as instances of quality data representations. Figure 9 shows correspondences of data representation in the real world to instances based on ontology. In the figure, each slot has an instance of its role player as a “value.” Similarly, the data of subjective responses that are used for interpretation of the construct of boredom can be adequately tagged and managed on the basis of our ontology.

Once the data of subjective responses are dealt with as instances of a specific concept in our ontology, the meanings of the data will be clear through the ontology. In this example, one of their meanings is which construct of boredom the relationships among the data that fill variables in the statistical model identify. Because an instantiated quality data representation can become a “value” of an instance of the “quality data” slot in the *Construct of Academic Boredom in Under-challenging situations* or the *Construct of Academic Boredom in Over-challenging situations*, the quality data slot is originally defined in the “mathematical model” (shown in Fig. 5). Thus, all the instances of quality data representations are linked with model formula slots defined in each node of the construct of boredom, which illustrates the classification of measured qualities.

**Fig. 10:**
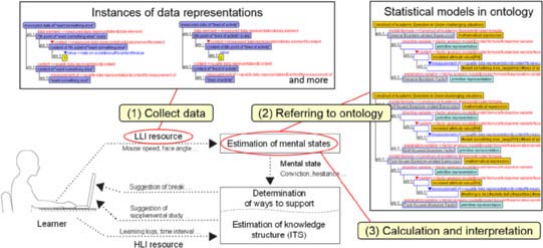
Procedure to interpret mental states using ontology in IMS

Management of linkages between data representations and construct boredom mentioned previously helps to interpret the construct of boredom for realizing a mentoring function of IMS. The concrete procedure of interpretation is as follows. First, IMS collects instances of data representation corresponding to each ABS-10. Second, IMS sorts the instances according to ontological descriptions, making groups in line with the class constraint of the “measurement of” slot in each construct. Third, IMS calculates ratios of TSS and compares them (a way mentioned in the previous subsection). Figure 10 shows these steps positioned in the skeleton of the IMS. In practical settings, instances of data representation are replaced with instances corresponding to estimated values from LLI resource data in the first step, and the IMS can adopt a more complex way to handle data in the third step, allowing the IMS to treat LLI resource data and mental states seamlessly. That is, the ontological description would be able to assist with the identification of the type of boredom, but only after estimation of the subjective response by using LLI resource data. Because estimation of the subjective response from LLI resource data heavily depends on individual learners, the construct of boredom represented as a statistical model is less useful for directly identifying emotions from granular data such as LLI resource data. However, as such, an ontology in which the constructs of emotions are implemented is helpful for obtaining an integrated understanding of learners, after estimating and identifying the values to be assigned to variables used in statistical models. Thus, our ontological descriptions have utility for developing IMS and similar systems with mentoring functions.

However, this demonstration does not exploit the capability of the descriptions of statistical models, because descriptions of qualities measured by items in the ABS lack sophistication. In concrete terms, qualities measured by ABS such as “want something else” and “tired of activity” are merely named in line with the abbreviation of the question items. These qualities may or may not expose essential natures in other studies of psychology. Thus, we analyze and extract the nature of the qualities by collecting other research pertaining to construct of academic boredom in future work.

## Conclusion

This study conceptualized three features of the boredom construct derived from the factor analysis conducted by Acee et al. (2010). By instantiation of data representation measured by the ABS-10, we demonstrated procedures to interpret the construct of boredom by IMS and similar systems with mentoring functions and yielded utility of the ontological descriptions. However, some issues remain. That is, descriptions of qualities measured by items in the ABS lack sophistication. In this study, we provided an adequate description of relationships between modeled attribute and quality values measured with rating scales. Moreover, we addressed modeled values described by the rating scales and offered tentative descriptions of the constructs of academic boredom and positioned them in the statistical models. However, the concepts related to these constructs fundamentally differ from general statistical models. In other words, the constructs should be conceived in ways similar to learner models. This is a topic that we hope to examine in the future.

Our ontology will enable researchers to better interpret their results and share their findings. The descriptions of constructs of academic boredom provided can help researchers acquire knowledge about associations between academic emotions and psychological attributes. Because the descriptions provided in the current study derive from a single study only, their capability and range of application are confined to the construct of academic boredom from the viewpoint of few researchers. However, basic forms of statistical models that represent the constructs of academic emotions are common in psychology. Thus, the current study simply proposed the descriptions as a framework of the knowledge sharing on academic emotions. In future work, we will extend our descriptions of constructs to include various academic emotions studied in educational psychology and conduct practical assessments of their validity and utility through an implementation of IMS.
